# Responses to terrestrial nest predators by endemic and introduced Hawaiian birds

**DOI:** 10.1002/ece3.6021

**Published:** 2020-01-23

**Authors:** George C. Cummins, Tad C. Theimer, Eben H. Paxton

**Affiliations:** ^1^ Department of Biological Sciences Northern Arizona University Flagstaff AZ USA; ^2^ Pacific Island Ecosystems Research Center U.S. Geological Survey Hawaii National Park HI USA

**Keywords:** introduced species, multipredator hypothesis, nest predation, novel predator, predator response

## Abstract

Birds free from nest predators for long periods may either lose the ability to recognize and respond to predators or retain antipredator responses if they are not too costly. How these alternate scenarios play out has rarely been investigated in an avian community whose members have different evolutionary histories. We presented models of two nest predators (rat and snake) and a negative control (tree branch) to birds on Hawaiʻi Island. Endemic Hawaiian birds evolved in the absence of terrestrial predators until rats were introduced approximately 1,000 years ago. Introduced birds evolved with diverse predator communities including mammals and snakes, but since their introduction onto the island approximately one century ago have been free from snake predation. We found that (a) endemic and introduced birds had higher agitation scores toward the rat model compared with the branch, and (b) none of the endemic birds reacted to the snake model, while one introduced bird, the Red‐billed Leiothrix (*Leiothrix lutea*), reacted as strongly to the snake as to the rat. Overall, endemic and introduced birds differ in their response to predators, but some endemic birds have the capacity to recognize and respond to introduced rats, and one introduced bird species retained recognition of snake predators from which they had been free for nearly a century, while another apparently lost that ability. Our results indicate that the retention or loss of predator recognition by introduced and endemic island birds is variable, shaped by each species' unique history, ecology, and the potential interplay of genetic drift, and that endemic Hawaiian birds could be especially vulnerable to introduced snake predators.

## Graphical abstract

Birds on islands without predators may lose appropriate predator responses and become vulnerable to extinction when predators are later introduced. By exposing native and non‐native Hawaiian birds to models of a predator never on Hawai‘i (snake) and a predator introduced 1,000 years ago (rat), we found that both native and introduced birds had evolved or retained response to the rat, but only one introduced bird had retained its response to the snake.

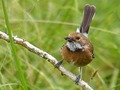

## 1 INTRODUCTION

Nest predation is the main cause of nest failure and therefore is one of the main drivers of life‐history traits and parental care behaviors in birds (Martin, 1995, 2015). In particular, the eggs and altricial young of passerine birds are highly vulnerable to predation, and most nests fail before eggs hatch or before the young leave the nest (Martin, Scott, & Menge, 2000). As a result, parental care behaviors should be under strong selection pressure to minimize the risk of predation. Evolutionary response to nest predation may influence reproductive traits, including nest placement, clutch size, length of nesting periods, and nestling growth rates (Conway & Martin, 2000; Martin, 2015; Martin et al., 2000). Parental care behaviors may also influence the level of predation at individual nests, either through parental activity that draws predators' attention or through defensive behaviors toward predators (Ghalambor, Peluc, & Martin, 2013; Halupka & Greeney, 2009; Martin et al., 2000; Skutch, 1949).

When birds have been free from nest predation, or from particular types of nest predators, for long periods, the ability of birds to recognize and respond to those nest predators may be diminished. The lack of appropriate antipredator behaviors can be especially detrimental when nest predators are introduced into areas where they had never been present historically (Griffin, Blumstein, & Evans, 2000). This is true for birds on many islands around the globe, and introduced predators are the main cause of extinction for many endemic island species (Blackburn, Cassey, Duncan, Evans, & Gaston, 2004; Savidge, 1987). In some cases, however, birds may learn appropriate responses to introduced predators (Griffin et al., 2000; Jamieson & Ludwig, 2012) or show plasticity in their response to predators depending on the level of nest predation by a certain predator type (Kleindorfer, Fessl, & Hoi, 2005; Peluc, Sillett, Rotenberry, & Ghalambor, 2008). For example, wild New Zealand Robins (*Petroica australis*) in New Zealand may learn from a single “training” encounter with an introduced predator, and respond more appropriately the next time they are faced with this threat (Maloney & McLean, 1995). This indicates that although many island birds have gone extinct after novel predator introductions, others may adapt to novel predators in evolutionarily short time frames.

Many island species are not entirely free of predation, but rather only free from certain predator types (e.g., an island may have avian predators but no mammalian predators). The presence of at least one type of predator may explain why some species adapt to novel predators over relatively short time scales. Griffin et al. (2000) proposed that species should learn to recognize and respond to a novel predator more easily if the novel predator used the same hunting technique as a current predator, because appropriate antipredator behaviors would already be in place. The multipredator hypothesis (Blumstein, 2006) goes further, proposing that antipredator behaviors are the result of pleiotropic genes that are linked and therefore will not be lost as long as some type of predation pressure exists, unless the relative costs of maintenance are too high. Under this hypothesis, species on islands without any predators may lose antipredator behaviors relatively quickly (Blumstein, Daniel, & Springett, 2004), while those that experience a limited predator suite should retain the ability to respond to both the predators present on the island and those that are subsequently introduced (Blumstein, 2006; Blumstein, Daniel, Griffin, & Evans, 2000). If a specific antipredator behavior has a high‐cost relative to other behaviors, the high‐cost behavior could be lost when predation pressure is removed (Blumstein, 2006).

Hawaiian forest bird communities, composed of native endemic and recently introduced non‐native species, offer an opportunity to experimentally study predator recognition and response in a community of birds with different evolutionary histories, on islands that were initially free of terrestrial predators and subsequently experienced introductions of additional predator types (i.e., mammalian predators). While avian predators such as the ‘Io (Hawaiian Hawk, *Buteo solitarius*) and Pueo (Short‐eared Owl, *Asio flammeus sandwichensis*) are endemic to Hawaiʻi, endemic Hawaiian birds evolved free from two of the most prominent nest predator types on the mainland (snakes and small mammals), as no terrestrial reptiles occurred on Hawaiʻi prior to human contact, and the only native terrestrial mammals were insectivorous bats (Pratt, 2009). Polynesians first arrived in the islands approximately 1,000 years ago (Wilmshurst, Hunt, Lipo, & Anderson, 2011) and brought with them the Polynesian rat (*Rattus exulans*), a mammal that has presumably acted as a nest predator since its introduction (Lindsey, Hess, Campbell, & Sugihara, 2009). Europeans made contact in 1778 and since then have introduced more terrestrial mammalian predators, including Javan mongooses (*Herpestes javanicus*), domestic cats (*Felis catus*), and two additional species of rats (Lindsey et al., 2009), including the arboreal black rat (*R. rattus*) that is known to be a particularly effective nest predator (Atkinson, 1977). Snakes, however, have not been established on any Hawaiian island. In addition, many non‐native passerine birds have been introduced to the islands, mostly in the last 100 years, and these mainland species have had long evolutionary histories with mammalian and reptilian nest predators. Given that these introduced species are now coexisting in native habitats with endemic Hawaiian birds (Foster, 2009), differences in their responses to simulated nest predators can provide insights into the evolution and maintenance of antipredator behavior in birds with different evolutionary histories.

The ability of a bird species to make an appropriate behavioral response to a predator depends on recognition of a predator as a threat. We designed a study to test three alternative hypotheses about the response of native and non‐native birds to an introduced predator (rat) and a predator that has never been present on the islands (snake). First, millions of years of isolation from rat and snake predators experienced by the descendants of the initial avian colonists to the islands could have led to the loss of recognition and response to terrestrial predators, and therefore, endemic species would not respond when faced with either a snake or a rat in an experimental trial, in spite of the potential for interaction with introduced rat predators over the last 1,000 years. Other island endemics have lost recognition of predators over time (Blackburn et al., 2004; Griffin et al., 2000), presumably because the costs associated with these behaviors were too high in the absence of predators (Griffin et al., 2000; Lima & Dill, 1990). Alternatively, because Hawaiian endemics have not lived entirely predator‐free, as there are endemic avian nest predators on the islands, the presence of these avian nest predators could have maintained antipredator behaviors that could now be expressed toward both mammalian and reptilian predators due to pleiotropic effects and/or relatively low maintenance costs of those behaviors (i.e., the multipredator hypothesis, Blumstein, 2006). Terrestrial, climbing nest predators rely on different hunting techniques than raptors and could elicit different responses when recognized by prey species (Muralidhar, 2017), though this does not always seem to be the case for nest defense (Patterson, Petrinovich, & James, 1980; White, 2014; see discussion in Muralidhar, 2017). A third alternative could be that recognition and response to snakes and rats has been lost in endemic birds over the long period free from these predator types, but those behaviors could have been re‐established toward rats through learning from direct experience, or population‐level selection. New Zealand passerine species have shown recognition and response toward introduced mammalian predators (Jamieson & Ludwig, 2012; Massaro, Starling‐Windhof, Briskie, & Martin, 2008; White, 2014) in spite of having evolved in the absence of mammalian predators, and O‘ahu ʻElepaio (*Chasiempis ibidis*) have shifted their nesting behavior in response to heavy rat predation (Vanderwerf, 2012). Under this hypothesis, endemic bird species would respond to rat models but would not generalize their recognition to another taxonomic group (e.g., reptiles; Ferrari, Gonzalo, Messier, & Chivers, 2007; Griffin, Evans, & Blumstein, 2001).

Non‐native birds, such as the Japanese White‐eye (*Zosterops japonicus*) and Red‐billed Leiothrix (*Leiothrix lutea*), were brought to Hawai‘i within the last century from mainland areas where both snake and rat predators were present. Given that rats were already established and numerous on Hawai‘i at the time non‐native bird species were introduced, we predicted that their antipredator response toward rats should be maintained. However, we hypothesized two alternative responses by introduced birds to snakes that are absent from Hawai‘i. First, given introduced birds have been living without snake predators on Hawai‘i for approximately 90 years (20–30 generations for a small passerine), this relatively short‐term absence of snake predators could cause loss of recognition and response to snake predators, especially if this is a learned trait with little or no innate component. Alternatively, if predator responses have a strong genetic component, the period since their introduction may not have been sufficiently long enough for loss of recognition and response toward snakes. This would be especially true if antipredator responses to rats have maintained a suite of responses broad enough to include snake predators (i.e., multipredator hypothesis, Blumstein, 2006). Understanding the patterns of predator responses in endemic and introduced birds with differing evolutionary histories is important in light of the increasing rate of species introductions around the world and the threatened and endangered conservation status of many endemic island birds (Seebens et al., 2017).

## 2 METHODS

### 2.1 Study area

We conducted our study within Hakalau Forest National Wildlife Refuge (NWR; hereafter Hakalau) on Hawaiʻi Island, Hawaiʻi from February through June in both 2015 and 2016. Hakalau is located on the windward slopes of Mauna Kea, and encompasses a broad elevation (793–2,000 m) and rainfall (254–635 cm annual precipitation) gradient, with lower elevations receiving more rain (USFWS, 2010). Hakalau largely consists of intact native ‘ōhi‘a (*Metrosideros polymorpha*)‐koa (*Acacia koa*) rainforest, and intense reforestation efforts have been conducted since 1989 to restore the remaining 1,620 ha of former pasture to native forest.

We used three previously established study sites during both years of data collection: Pedro (elevation 1,524 m), Koa Reforestation (elevation 1,585 m), and Pua Akala (elevation 1,890 m). The Pedro and Pua Akala sites were characterized by extensive groves of mature ‘ōhi‘a‐koa forest with areas of dense native understory interspersed with open areas of non‐native grasses. The Koa Reforestation site was an even‐aged, reforested stand of koa ca. 20 years old, with a closed canopy and little understory except non‐native grasses and recently planted native shrubs that have been recolonized by forest birds (Paxton et al., 2017).

### 2.2 Study species and nest finding

We experimentally tested predator response behavior at the nests of four endemic Hawaiian species that have been present on the islands for 1.5–6 million years: Hawai‘i ʻAmakihi (*Chlorodrepanis virens*) and ʻIʻiwi (*Drepanis coccinea*), Hawaiian honeycreepers (Fringillidae) whose ancestors arrived in Hawai‘i 6–7 million years ago (Lerner, Meyer, James, Hofreiter, & Fleischer, 2011); ‘Ōma‘o (*Myadestes obscurus*), a thrush (Turdidae) whose ancestors arrived in Hawai‘i 6–7 million years ago (Fleischer & McIntosh, 2001); and Hawai‘i ʻElepaio (*Chasiempis sandwichensis*), whose ancestors arrived in Hawai‘i circa 2.3 million years ago (VanderWerf, Young, Yeung, & Carlon, 2010). In addition, two introduced species, Japanese White‐eye and Red‐billed Leiothrix, were introduced within the last century. We found nests by visually searching the canopy and by following behavioral cues such as defensive displays toward humans or birds carrying nesting material. All species except the Red‐billed Leiothrix were also being color banded as part of a larger demographic project, allowing us to differentiate some individual pairs, although not all pairs used in this study included a color‐banded adult. This experiment took place during the mid‐ to late nestling period (nestling day 7 onward) to control for any potential changes in predator response associated with nestling developmental stage (Patterson et al., 1980).

### 2.3 Nest predator response

**Figure 1:**
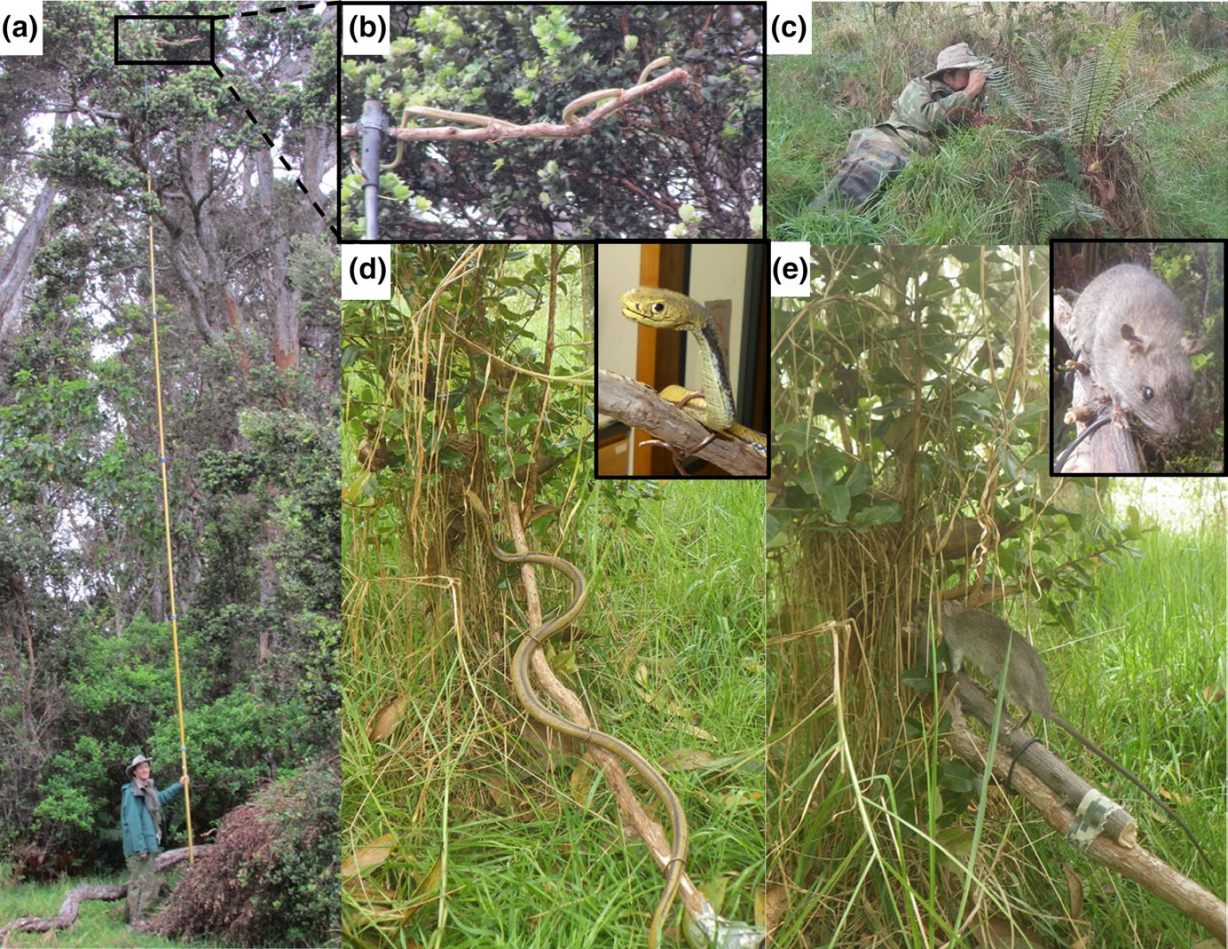
Methods used to conduct experimental behavioral trials at nests of six focal species of birds in Hakalau Forest NWR, Hawai‘i, USA in 2015 and 2016. (a) Predator models were placed on an ‘ōhi‘a branch and raised to the height of the nest on an extending pole, with (b) the model oriented with head toward the nest. (c) The observer then hid as far away from the nest as possible while still being able to observe the nest. (d) A realistic rubber snake and (e) mounted rat were used as predator models, and an ‘ōhi‘a branch alone was used as the negative control

We recorded responses by each study species to models of two predators: a rubber snake and a taxidermy mounted rat. We used two identical dull yellow and brown colored 122 cm long polyvinyl snakes, molded into a sinuous posture with head slightly raised (“yellow rat snake” purchased from http://www.veghead.com). The models resembled the Japanese rat snake (*Elaphe climacophora*). Two black rats were used as taxidermy mounts; one smaller and blacker and the other larger and greyer in color. Predator models were attached to a branch from the most common tree (‘ōhi‘a), and we also presented the branch alone at nests as a negative control, assuming the branch served as a familiar, nonthreatening object birds encounter daily (Figure 1). Our experimental protocol was to randomly choose one of the objects (rat, snake, or branch) and place it within 1 m of the nest on the first day of trials, followed by the remaining two objects (in random order) on subsequent days, so no nest experienced more than one trial per day. Because nests ranged from 1 to over 10 m above the ground, we mounted each model on the 1 m ‘ōhi‘a branch and attached this to a 12 m telescoping pole painted with a camouflage pattern on the upper 4 m of the pole. At each nest, we waited until neither adult was present before raising the predator model to the nest with the model's head oriented at the nest, then propped the pole in place by leaning it against branches or the trunk of the tree, and retreated to an observation point. The observer hid among vegetation at least 10 m away (and often farther) while still having a view of the nest area. We recorded all trials using video cameras placed approximately 10–20 m from the nest (Pentax Optio WG‐2, Ricoh Imaging Company, LTD and Panasonic Lumix FZ200, Panasonic Corporation) and hand‐held voice recorders. We started each trial when one bird approached within 2 m of the nest or model and the trial lasted for the following 5 min. The first member of the pair of birds associated with the nest to enter this 2 m zone was considered the focal bird for observations of calls and movements; however, behaviors like feeding nestlings or attacking models by either pair member were noted. After the 5‐min trial, we removed the model and pole from the area.

We quantified each individual bird's predator response behaviors by recording the number of call notes and the number of movements made by the focal bird, and whether either parent fed the nestlings during the trial (Cummins, Theimer, & Paxton, 2019). We used the number of calls and movements as response variables because alarm calls and distraction movements are widespread antipredator behaviors for passerines (Curio, 1988; Montgomerie & Weatherhead, 1988), and birds are not likely to feed nestlings if they perceive a nearby nest predator. For the endemic species, we counted the number of individual call notes, but for Red‐billed Leiothrix and Japanese White‐eye we recorded total time calling because they do not utter single notes, but rather string together long bouts of chattering when agitated. We defined a movement as the bird moving to a different physical location (e.g., hopping from one branch to another or flying from one side of the tree to the other), but did not include movements that did not change a bird's physical location (e.g., a bird turning around in place on a branch). We restricted behavioral observations (e.g., the number of calls and movements) to 5 m or less from the nest, as birds typically could not be seen when farther than this distance from the nest.

### 2.4 Statistical methods

Because each species has inherently different levels of response to predators, we normalized movement and call rates in a given trial as a percentage of the maximum recorded response across all trials for a given species to facilitate comparison among species. For example, for a trial using the rat model at a Japanese White‐eye nest, the number of movements observed in that trial was divided by the maximum number of movements ever recorded in a Japanese White‐eye trial with any model. We then used a principle components analysis (PCA) to combine the three behavioral responses into an overall agitation score using the first principle component (PC1), the component that explains most of the variation. PCA was done using a singular value decomposition method, centered and scaled, with the “stats” package in Program R, version 3.5 (R Core Team, 2018). We used linear mixed‐effects models (“lme4” package version 1.1 in Program R; Bates, Maechler, Bolker, & Walker, 2015) to determine if the agitation score differed between predator model types within each species. The agitation score was the response variable, the predator model type was the independent variable, and nest ID was used as the random effect. Pairwise comparisons were made between groups using Tukey's honest significant differences test (Zar, 2009). Statistical significance was accepted at the *α* = .05 level.

## 3 RESULTS

Over the two field seasons, we conducted 136 trials from 57 nests across the six species (all three predator models were not successfully presented at every nest, so the number of trials is not three times the number of nests). Sample sizes were larger in the introduced species (19 nests for the Japanese White‐eye, 16 nests for the Red‐billed Leiothrix) compared with the endemic species (10 nests for Hawai‘i ‘Elepaio, seven nests for ʻŌmaʻo, three nests for ‘I‘iwi, and two nests for the Hawai‘i ‘Amakihi). We combined the trial data for the four endemic species for formal analysis, due to (a) overall similar behavioral responses by all these species to the predator models, (b) relative rarity of accessible nests of endemic species relative to non‐natives, and (c) because our overarching goal was to compare behavioral responses of birds isolated from predators for millions of years to those of recently introduced species. In over half the trials, the gender of the focal bird could not be determined. In the remaining trials, similar numbers of each sex acted as the focal bird (35 trials for the male and 31 trials for the female), and both members were sampled during different trials at 11 nests. We used PCA analysis to combine the three behavioral responses (movements, number of calls, and nestling feeding) into a single overall “agitation score” (PC1) for our analyses of bird response to predators with PC1 accounting for 74% of the total observed variance. Only complete trials were used for PCA analysis (i.e., trials with all three behavior metrics successfully quantified), which resulted in a sample size of 117 trials from 54 nests (removing two Japanese White‐eye and one Red‐billed Leiothrix nests from the above nest totals). All complete individual trials were used in the PCA analysis, even if all three predator models were not successfully presented to each of these nests.

**Figure 2:**
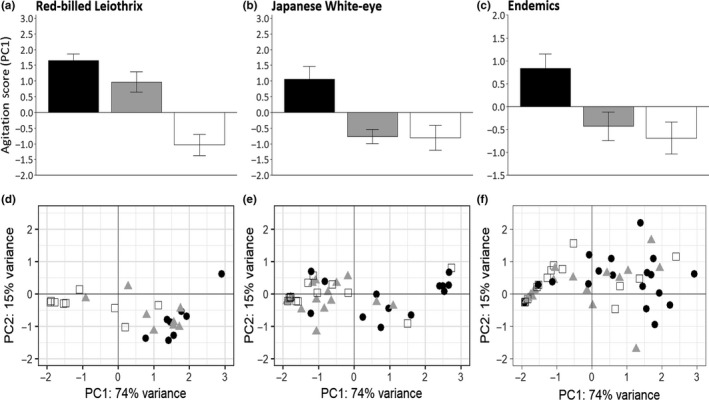
The top row (a–c) shows the mean and *SE* of agitation scores (PC1) of (a) introduced Red‐billed Leiothrix, (b) introduced Japanese White‐eye, (c) endemic Hawaiʻi passerines combined (Hawaiʻi ʻElepaio, ʻŌmaʻo, Hawaiʻi ʻAmakihi and ‘Iʻiwi) when presented with a rat model (black), a snake model (gray), or a branch (white). The bottom row (d–f) shows the first (PC1) versus the second (PC2) principal components to illustrate the variation in response by the (d) introduced Red‐billed Leiothrix, (e) introduced Japanese White‐eye, (f) endemic Hawaiʻi passerines combined (Hawaiʻi ʻElepaio, ʻŌmaʻo, Hawaiʻi ʻAmakihi and ‘Iʻiwi) to each predator model (black circles = Rat, gray triangles = Snake, open squares = Branch), during experimental trials in Hakalau Forest NWR, Hawai‘i, USA in 2015 and 2016. The relative distance between points indicates the similarity/dissimilarity of behavioral responses in the various experimental trials

The non‐native Japanese White‐eye and the endemic birds were similar in their behavioral responses (Figure 2). Both the Japanese White‐eye and the endemics had a significantly higher agitation score for the rat compared with the snake and the branch, while the response to snake and branch did not differ significantly: linear mixed‐effects model for Japanese White‐eye: *F*_2_ = 9.72, *p* < .001; pairwise differences using Tukey method: rat versus snake *p* = .002, rat versus branch *p* = .002, snake versus branch *p* = .998 (Figure 2b); linear mixed‐effects model for endemics: *F*_2_ = 8.91, *p* < .001; pairwise differences using Tukey method: rat versus snake *p* = .006, rat versus branch *p* = .002, snake versus branch *p* = .753 (Figure 2c). In both the Japanese White‐eye and the combined endemics, the number of movements and calls during a trial was 1.5–2.5 times higher toward the rat model compared with the other two models, and in both cases birds fed their nestlings less during rat trials than when the other two models were present (Table 1). Although this overall pattern was evident in all the endemic species individually, there was greater variation, both among individuals in a species and among species, as evident in PC2 (Figure 2f).

**Table 1:** Number of unique nests and relativized mean (±*SE*) for the number of movements and calls, and the percent of trials within each predator model type where adults did not feed their nestlings during experimental trials with two predator models (rat and snake) and one control model (branch) for two introduced bird species (Red‐billed Leiothrix and Japanese White‐eye) versus an endemic group including Hawaiʻi ʻAmakihi, ʻIʻiwi, ʻŌmaʻo, and Hawaiʻi ʻElepaio at Hakalau Forest NWR, Hawai‘i, USA in 2015 and 2016

Species	Behavior	*N*	Mean ± *SE*		
			Rat	Snake	Branch
Red‐billed Leiothrix	Movements	16	46.0 ± 8.9	37.5 ± 3.6	14.6 ± 4.6
	Calls		91.4 ± 2.7	71.3 ± 7.7	23.0 ± 9.8
	Did not feed nestlings		100%	75.0%	20.0%
Japanese White‐eye	Movements	19	50.2 ± 8.1	25.0 ± 4.4	25.3 ± 7.0
	Calls		68.1 + 8.2	30.2 + 4.8	25.6 + 8.3
	Did not feed nestlings		68.8%	11.8%	13.3%
Endemics	Movements	22	58.6 ± 6.1	34.9 ± 6.2	32.4 ± 7.0
	Calls		42.6 ± 8.3	20.1 ± 6.1	13.4 ± 5.5
	Did not feed nestlings		73.7%	44.4%	26.7%

In contrast, the Red‐billed Leiothrix had significantly higher agitation scores for both the rat and the snake models compared with the control branch, while the scores toward rat and snake were not significantly different from each other (linear mixed‐effects model: *F*_2_ = 35.51, *p* < .001; pairwise differences using Tukey method: rat vs. snake *p* = .244, rat vs. branch *p* < .001, snake vs. branch *p* < .001; Figure 2a). The number of movements made during a trial was over two to three times higher toward these models than toward the branch (Table 1). Likewise, the amount of calls recorded was three to four times higher toward the snake and rat models. Red‐billed Leiothrix also did not feed their nestlings during any rat trial, nor at 75% of the snake trials.

In addition to the calls, movements and feeding behaviors that we quantified for our analyses, we noted other behaviors consistent with birds recognizing the models as threats rather than simply as novel stimuli. Some individuals, especially Red‐billed Leiothrix, oriented toward the predator model's head during their response. We also witnessed two pairs of ʻŌmaʻo physically attack the rat model, while ignoring the branch and snake models. In these latter two cases, both individuals of the pair were present, and stood upright, erecting the feathers on the breast, while fanning their tails and wings outward. They would then lunge downward at the rat until their beak contacted the rat's head. There were also several instances of Hawaiʻi ʻElepaio flying toward, and then hovering near, the model, but never physically touching it. None of these behaviors were seen during trials with the branch alone.

## 4 DISCUSSION

The Hawaiian avian community today is a diverse assemblage of both endemic and introduced species, presenting an opportunity to understand how behavioral responses evolve in novel systems. The endemic species have descended from a few lineages that colonized the Hawaiian archipelago over millions of years, from 5 to 6 million years for the honeycreepers and thrushes to 2.3 million years for the monarch flycatchers (Pratt, 2009). These species have evolved over millions of years in isolation from mammalian and reptilian predators, but have recently been exposed to mammalian predators (particularly the black rat). In contrast, recently introduced passerines have largely come from continental or large island systems that have diverse predator communities (Foster, 2009). By challenging this diverse community to nest predators both present and absent from Hawai‘i today, we were able to measure the range of responses and evaluate different hypothesis about antipredator behavior.

The two introduced bird species we studied exhibited strikingly different responses to the snake model, each providing responses more consistent with different hypotheses for the maintenance or loss of antipredator behaviors. The Red‐billed Leiothrix reacted strongly to both a current predator, the rat, and a historical predator, the snake, consistent with the prediction of the multipredator hypothesis (Blumstein, 2006). Because Red‐billed Leiothrix have experienced continuous threats from small mammal predators both in their natural range and after introduction to Hawai‘i, the multipredator hypothesis predicts they would retain recognition and responses to snakes, another climbing, terrestrial predator, even after living in Hawaiʻi without snakes for over 90 years. In this case, both predators were terrestrial, and our results suggest that recognition and response to snake predators in Red‐billed Leiothrix may be innately linked to behavioral responses toward small mammalian predators. Interestingly, Red‐billed Leiothrix on Hawai‘i have also retained responses to avian brood parasites (cuckoos) similar to that of populations on mainland China, even though avian brood parasites have never occurred on Hawai‘i (Yang, Liu, Zeng, & Liang, 2014). In contrast, introduced Japanese White‐eyes recognized and responded to the rat, but not to the snake, despite both bird species being introduced to Hawaiʻi at approximately the same time and therefore free of snake predators for similar periods. Instead, responses of the Japanese White‐eye were consistent with the hypothesis that even a relatively short time of relaxed selection pressure from snakes was enough for recognition and response toward this predator to be lost, a finding consistent with other studies that have documented rapid loss of predator recognition in the absence of predators (Jamieson & Ludwig, 2012; White, 2014). Snakes are also not present in New Zealand, and a similar nest predator response study found that introduced bird species did not respond to rubber snake models, while they did respond to predator models of endemic and introduced predators, similar to our result with Japanese White‐eye, and differing from that of Red‐billed Leiothrix (White, 2014). In a concurrent demographic study using these same study sites and individuals, we found nest predation rates to be quite low for endemic species (5% of known fate nests, ranging from zero at ʻIʻiwi nests to 23% of ʻŌmaʻo nests) and Japanese White‐eyes (14% of known fate nests), and higher for Red‐billed Leiothrix (35% of known fate nests; Cummins, Kendall, & Paxton, 2014). The relatively lower nest predation rate of Japanese White‐eye compared with Red‐billed Leiothrix should correspond to lower selection pressures and may help explain why Japanese White‐eye exhibited more variation in their recognition and response toward the predator models.

We assume that both species experienced nest predation by both rats and snakes in their ancestral ranges, although the source populations for the two introduced species is somewhat ambiguous, and therefore, it is difficult to know the level and type of nest predation their ancestral populations faced (Foster, 2009; Pyle & Pyle, 2009). The current assumption is that the Red‐billed Leiothrix was introduced from populations in south China and possibly Nepal, while the Japanese White‐eye was introduced from mainland Japan (Pyle & Pyle, 2009). There are snakes in both locations, but it is possible that the sources from which Japanese White‐eyes were drawn may have been areas free from snake predators. Alternatively, founder effects and genetic drift in small, isolated island populations after colonization could have caused loss of antipredator behaviors in Japanese White‐eyes but not Red‐billed Leiothrix (Blumstein & Daniel, 2005). If this is the case, our results suggest that any relationship between time away from predators and the loss of responses toward those predators may vary from species to species.

Of the three hypotheses, we posited about how endemic Hawaiian birds might respond to predators, the one most consistent with our findings was that recognition and response to snakes and rats was lost in the long period without these predator types, but those behaviors were re‐established toward rats through recent experience or selection. These endemic species represented three different avian families that evolved for millions of years in the absence of both predators and in all three, some individuals now show a response to rat models, but not to snake models. This is a strikingly similar result to that found with endemic New Zealand birds, which responded to endemic and introduced predator models, but not to snakes (White, 2014). If, instead of loss and reacquisition, ancestral antipredator responses had been retained over millions of years, we would have expected similar responses to both predator types. Some species of passerine birds have been shown to learn responses to novel predators in evolutionarily short time frames (Griffin et al., 2000; Maloney & McLean, 1995; Massaro et al., 2008), while others appear to have innate responses to novel predators (Veen, Richardson, Blaakmeer, & Komdeur, 2000). Mammals have also shown the ability to respond appropriately to novel predators after evolutionary short time frames (Bytheway & Banks, 2019), indicating this phenomenon is not unique to one taxonomic lineage. Our study cannot distinguish whether the endemic birds we studied had learned to recognize rats based on direct experience or whether an innate, genetically based response has been selected for over the last few hundred years. The greater variation among endemic birds in their response to the rat model would be consistent with either variation in individual experience with rats among individuals or with the relatively recent establishment of genes for recognition and response that had not yet spread throughout the population. This individual variation in response was best represented by endemic ʻŌmaʻo. In two cases, ʻŌmaʻo physically attacked the rat, while both individuals of another pair directed no activity toward the model and fed their nestlings with the rat model present. Likewise, in two separate ʻŌmaʻo trials using the rat, the male fed nestlings while the female did not.

Assuming the ancestors of endemic Hawaiian birds were able to recognize rats and snakes as predators, the loss of recognition of those predators occurred even though other predator types were present on the islands, indicating these responses may not follow the multipredator hypothesis. Prior to human contact, there were multiple endemic avian predators on Hawaiʻi, with two, the ‘Io and Pueo, still extant. Both species were observed on our study sites during the study, and multiple instances of ‘Io depredating nests were documented (G. Cummins, personal observation). The hunting method of these raptors is much different than that of the climbing terrestrial nest predators that our models represented. Birds of prey rely on vision to hunt instead of other senses like olfaction and can attack from a much greater distance. These endemic raptors eat adult and juvenile passerines as well as nestlings (Klavitter, 2009; Mounce, 2008; G. Cummins, personal observation), and the antipredator response toward them is different than toward smaller, terrestrial predators that are easier to evade. Endemic Hawaiian honeycreepers respond to ‘Io and Pueo by leaving the nest, freezing, and sometimes uttering alarm calls (Mounce, 2008; G. Cummins, personal observation), and Hawaiʻi ʻElepaio have been observed making alarm calls and flying agitatedly after owls that were in the vicinity of their nests (G. Cummins, personal observation), and mobbing ‘Io (VanderWerf, 2018). Although the multipredator hypothesis predicts antipredator responses to be conserved over time if the prey species still experiences some predation due to the genetic linkage of those behaviors (Blumstein, 2006), our results suggest the response to avian and terrestrial predators in endemic Hawaiian birds are not linked. This may be explained by the “predator archetype” hypothesis (Cox & Lima, 2006), in which avian and terrestrial predators are of different archetypes, and responses are not transferred from one predator type to another. If the predator archetype is a general phenomenon, it could explain why birds on many islands have shown naivete toward introduced mammals and reptiles even when endemic avian predators were present (Blackburn et al., 2004; Blumstein, 2006; Savidge, 1987).

How appropriate responses to novel predators have arisen in our system remains unknown. Passerines have been found to culturally transmit novel predator recognition through mobbing behaviors (Curio, 1988) and predator‐naïve New Zealand Robins needed only one exposure to simulated mobbing behavior of a predator to subsequently recognize and respond appropriately to that predator (Maloney & McLean, 1995). Variation in the probability of transmission of predator recognition through mobbing could potentially have contributed to the variation among species in our study. Although we recorded mobbing behavior in all 12 rat trials with Red‐billed Leiothrix and eight out of 16 rat trials for Japanese White‐eyes, we recorded mobbing in only six out of 19 rat trials with endemic birds (ʻElepaio were the native species most often observed mobbing in our study). Non‐native Japanese White‐eyes have been observed surrounding a perched ‘Io and giving alarm calls (Clarkson & Laniawe, 2000) and endemic ʻElepaio have been documented mobbing ‘Io (VanderWerf, 2018). If mobbing is an important behavior for culturally transmitting predator recognition, then a lack of this behavior should slow transmission. The lower mobbing rates by endemic species in our study is consistent with some historical observations (Perkins, 1903) and suggest that it may be less likely for endemic Hawaiian species to learn to recognize new predators through that mechanism.

Overall, our results suggest that introduced bird species may be better able to withstand introduction of novel predators than endemic birds, because introduced species are more likely to exhibit recognition and response to predators due to their more recent evolutionary history with those predators, but that snake predators may pose a greater risk than mammalian predators. Rats are believed to have had profoundly negative impacts on native bird species historically (Atkinson, 1977), but our study and others (VanderWerf, 2012; White, 2014) suggest native species have begun evolving responses to rats. However, the lack of response to a snake model by both endemic and one introduced bird species suggests that birds would be highly susceptible to introduced snake predators, such as the Brown Treesnake (*Boiga irregularis*) that extirpated many of Guam's native birds (Savidge, 1987). Given the devastating effect introduced snakes have had on the avifauna of other islands, and the naivety to snakes we documented, biosecurity efforts to prevent future introductions of snakes are warranted as a key part of conservation strategies for Hawai‘i's birds.

## CONFLICT OF INTEREST

The authors declare no conflict of interest.

## AUTHOR CONTRIBUTIONS

GCC, TCT, and EHP conceived and planned the experiments. GCC carried out the experiments and initial analyses. GCC, TCT, and EHP contributed to the interpretation of the results. GCC wrote the initial draft with modification by TCT and EHP. All authors helped shape the research, analyses, and interpretation.

## Data Availability

Behavioral response data for each of the predator trials are available at https://doi.org/10.5066/P9ADI937 (Cummins et al., 2019).
